# Supplementary material for: Changes in the Diversity and Composition of Gut Microbiota of Red-Crowned Cranes (Grus japonensis) after Avian Influenza Vaccine and Anthelmintic Treatment
Source: Animals (Basel). 2022 May 5;12(9):1183. doi: 10.3390/ani12091183 (PMC9099658; doi:10.3390/ani12091183)
Supplement: Supplementary file 1 [file animals-12-01183-s001.zip › animals-1697015-supplementary.pdf]

Table S1. Statistical table of sequencing quantity per sample.

Sample ID	Input	Filtered	Denoised	Merged	Nonchimeric
S1-B	66090	61385	60512	59336	55039
S2-B	56927	52256	50659	47199	30106
S3-B	64768	59907	57461	52469	31356
S1-D0	82866	77221	76679	75865	59405
S2-D0	73779	67862	67307	66333	45777
S3-D0	82779	77695	77178	76494	73802
S1-D5	62133	57789	56211	53852	39510
S2-D5	47053	42783	42006	40766	28707
S3-D5	78974	72662	70570	67083	57491
S1-D10	53346	48854	46571	41647	33077
S2-D10	48634	43791	41193	36610	28698
S3-D10	78035	72007	70780	68772	58834
S1-D15	44107	39801	38035	34653	25685
S2-D15	51557	46265	42411	34558	27778
S3-D15	65412	57690	55131	50715	43858

Table S2. Bray-curtis distance matrix for NMDS analysis.

Sample ID	S1-B	S2-B	S3-B	S1-D0	S2-D0	S3-D0	S1-D5	S2-D5	S3-D5	S1-D10	S2-D10	S3-D10	S1-D15	S2-D15	S3-D15
S1-B	0.00	0.84	0.97	0.99	0.98	1.00	0.99	0.98	0.99	0.99	0.93	1.00	1.00	0.99	0.99
S2-B	0.84	0.00	0.83	0.99	0.95	0.99	0.97	0.94	0.99	0.97	0.90	0.99	1.00	0.98	0.98
S3-B	0.97	0.83	0.00	0.85	0.90	0.84	0.85	0.86	0.82	0.85	0.90	0.82	1.00	0.96	0.80
S1-D0	0.99	0.99	0.85	0.00	0.36	0.68	0.59	0.73	0.80	0.60	0.92	0.67	1.00	0.99	0.76
S2-D0	0.98	0.95	0.90	0.36	0.00	0.77	0.62	0.62	0.85	0.60	0.84	0.75	1.00	0.99	0.82
S3-D0	1.00	0.99	0.84	0.68	0.77	0.00	0.72	0.82	0.41	0.77	0.92	0.27	1.00	0.99	0.46
S1-D5	0.99	0.97	0.85	0.59	0.62	0.72	0.00	0.68	0.77	0.63	0.82	0.70	1.00	0.86	0.73
S2-D5	0.98	0.94	0.86	0.73	0.62	0.82	0.68	0.00	0.85	0.78	0.78	0.81	1.00	0.98	0.82
S3-D5	0.99	0.99	0.82	0.80	0.85	0.41	0.77	0.85	0.00	0.77	0.90	0.33	1.00	0.95	0.35
S1-D10	0.99	0.97	0.85	0.60	0.60	0.77	0.63	0.78	0.77	0.00	0.82	0.74	0.98	0.88	0.77
S2-D10	0.93	0.90	0.90	0.92	0.84	0.92	0.82	0.78	0.90	0.82	0.00	0.91	0.85	0.88	0.88
S3-D10	1.00	0.99	0.82	0.67	0.75	0.27	0.70	0.81	0.33	0.74	0.91	0.00	1.00	0.98	0.35
S1-D15	1.00	1.00	1.00	1.00	1.00	1.00	1.00	1.00	1.00	0.98	0.85	1.00	0.00	0.88	1.00
S2-D15	0.99	0.98	0.96	0.99	0.99	0.99	0.86	0.98	0.95	0.88	0.88	0.98	0.88	0.00	0.94
S3-D15	0.99	0.98	0.80	0.76	0.82	0.46	0.73	0.82	0.35	0.77	0.88	0.35	1.00	0.94	0.00

Table S3. Statistics of the number of microbial taxa at the phylum and genus levels.

Taxonomic level	Sample ID	S1-B	S2-B	S3-B	S1-D0	S2-D0	S3-D0	S1-D5	S2-D5	S3-D5	S1-D10	S2-D10	S3-D10	S1-D15	S2-D15	S3-D15
Phylum	Firmicutes	7.30%	57.77%	71.17%	99.21%	99.27%	98.64%	85.90%	94.88%	92.66%	59.26%	43.51%	96.41%	55.80%	38.38%	79.09%
	Proteobacteria	4.58%	15.03%	20.91%	0.19%	0.20%	0.36%	7.00%	1.89%	4.21%	25.91%	30.04%	2.19%	33.58%	31.33%	14.35%
	Fusobacteria	86.35%	22.71%	0.00%	0.00%	0.07%	0.01%	0.02%	0.01%	0.12%	0.00%	5.62%	0.00%	0.02%	0.10%	0.00%
	Actinobacteria	0.46%	0.90%	6.78%	0.40%	0.20%	0.58%	4.28%	1.78%	1.21%	8.46%	7.35%	0.56%	8.60%	18.00%	3.98%
	Bacteroidetes	0.21%	1.88%	0.00%	0.02%	0.02%	0.01%	0.00%	0.00%	0.10%	0.04%	5.19%	0.01%	0.18%	0.05%	0.11%
	Chloroflexi	0.00%	0.01%	0.32%	0.01%	0.00%	0.00%	0.22%	0.13%	0.27%	0.39%	0.68%	0.10%	0.16%	4.38%	0.49%
	Tenericutes	0.00%	0.00%	0.00%	0.00%	0.06%	0.11%	0.86%	0.04%	0.27%	0.30%	1.45%	0.11%	0.00%	2.83%	0.00%
	Cyanobacteria	0.01%	0.31%	0.08%	0.01%	0.00%	0.00%	0.32%	0.00%	0.02%	3.30%	0.12%	0.00%	0.06%	0.14%	0.01%
	[Thermi]	0.04%	0.01%	0.00%	0.00%	0.02%	0.00%	0.07%	0.02%	0.00%	0.01%	2.57%	0.00%	0.00%	0.00%	0.00%
	TM7	0.00%	0.00%	0.03%	0.00%	0.00%	0.02%	0.05%	0.03%	0.20%	0.16%	0.19%	0.07%	0.02%	0.48%	0.58%
	Others	1.05%	1.37%	0.71%	0.17%	0.17%	0.27%	1.29%	1.22%	0.94%	2.16%	3.28%	0.54%	1.58%	4.31%	1.39%
Genus	*Lactobacillus*	1.40%	4.56%	20.47%	98.19%	85.09%	96.70%	65.70%	61.03%	79.60%	49.27%	15.22%	88.88%	0.20%	0.68%	70.49%
	Cetobacterium	85.78%	10.26%	0.00%	0.00%	0.00%	0.01%	0.02%	0.01%	0.12%	0.00%	5.56%	0.00%	0.00%	0.10%	0.00%
	Sporosarcina	0.00%	0.11%	0.27%	0.02%	0.00%	0.09%	0.01%	0.10%	0.17%	0.44%	7.07%	0.10%	34.17%	10.28%	0.30%
	Lactococcus	0.01%	0.00%	0.11%	0.06%	0.11%	0.10%	13.64%	0.08%	4.27%	5.55%	3.05%	3.60%	0.03%	17.35%	2.63%
	Acinetobacter	0.02%	0.00%	0.00%	0.00%	0.00%	0.00%	0.68%	0.00%	1.65%	0.70%	2.17%	0.78%	0.04%	2.28%	8.53%
	Paracoccus	0.08%	0.00%	0.02%	0.00%	0.00%	0.01%	1.69%	0.10%	0.11%	1.41%	3.68%	0.04%	4.58%	4.29%	0.17%
	Clostridiaceae_Clostridium	0.77%	3.48%	6.75%	0.00%	0.02%	0.01%	0.01%	0.09%	0.44%	0.02%	0.30%	0.23%	1.26%	0.82%	0.41%
	Agrobacterium	1.07%	2.55%	6.04%	0.00%	0.00%	0.00%	0.09%	0.00%	0.04%	0.39%	0.06%	0.00%	0.00%	1.02%	0.13%
	Turicibacter	0.00%	3.59%	6.98%	0.01%	0.00%	0.00%	0.00%	0.00%	0.00%	0.00%	0.00%	0.00%	0.00%	0.00%	0.00%
	Campylobacter	0.21%	8.13%	0.02%	0.00%	0.00%	0.00%	0.00%	0.00%	0.00%	0.00%	0.04%	0.00%	0.00%	0.01%	0.00%
	Nocardioides	0.01%	0.00%	0.91%	0.11%	0.00%	0.00%	1.45%	0.19%	0.16%	1.75%	0.70%	0.11%	0.05%	1.41%	0.78%
	Oceanisphaera	0.01%	0.00%	0.00%	0.00%	0.00%	0.00%	0.00%	0.01%	0.00%	0.05%	2.92%	0.00%	3.79%	0.05%	0.00%
	Zobellella	0.00%	0.00%	0.00%	0.00%	0.00%	0.00%	0.00%	0.00%	0.00%	0.01%	1.44%	0.00%	5.20%	0.00%	0.00%
	[Clostridium]	0.15%	1.34%	4.18%	0.03%	0.00%	0.00%	0.00%	0.10%	0.03%	0.00%	0.40%	0.00%	0.00%	0.09%	0.02%
	Arthrobacter	0.01%	0.00%	0.13%	0.02%	0.00%	0.07%	0.47%	0.03%	0.10%	1.32%	1.40%	0.03%	1.47%	0.59%	0.30%
	Others	10.49%	65.98%	54.13%	1.56%	14.76%	2.99%	16.23%	38.27%	13.32%	39.10%	55.97%	6.23%	49.21%	61.05%	16.23%
